# Discovery of *Mycobacterium tuberculosis* Protein Tyrosine Phosphatase B (PtpB) Inhibitors from Natural Products

**DOI:** 10.1371/journal.pone.0077081

**Published:** 2013-10-14

**Authors:** Alessandra Mascarello, Mattia Mori, Louise Domeneghini Chiaradia-Delatorre, Angela Camila Orbem Menegatti, Franco Delle Monache, Franco Ferrari, Rosendo Augusto Yunes, Ricardo José Nunes, Hernán Terenzi, Bruno Botta, Maurizio Botta

**Affiliations:** 1 Laboratório Estrutura e Atividade, Universidade Federal de Santa Catarina, Florianópolis, Brazil; 2 Dipartimento di Chimica e Tecnologie del Farmaco, Università di Roma La Sapienza, Roma, Italy; 3 Dipartimento Farmaco Chimico Tecnologico, Università degli Studi di Siena, Siena, Italy; 4 Centro de Biologia Molecular Estrutural, Universidade Federal de Santa Catarina, Florianopolis, Brazil; 5 Center for Biotechnology, Sbarro Institute for Cancer Research and Molecular Medicine, Temple University, Philadelphia, Pennsylvania, United States of America; Institute of Molecular Genetics IMG-CNR, Italy

## Abstract

Protein tyrosine phosphatase B (PtpB) is one of the virulence factors secreted into the host cell by *Mycobacterium tuberculosis*. PtpB attenuates host immune defenses by interfering with signal transduction pathways in macrophages and, therefore, it is considered a promising target for the development of novel anti-tuberculosis drugs. Here we report the discovery of natural compound inhibitors of PtpB among an *in house* library of more than 800 natural substances by means of a multidisciplinary approach, mixing *in silico* screening with enzymatic and kinetics studies and MS assays. Six natural compounds proved to inhibit PtpB at low micromolar concentrations (< 30 µM) with Kuwanol E being the most potent with *K*_i_ = 1.6 ± 0.1 µM. To the best of our knowledge, Kuwanol E is the most potent natural compound PtpB inhibitor reported so far, as well as it is the first non-peptidic PtpB inhibitor discovered from natural sources. Compounds herein identified may inspire the design of novel specific PtpB inhibitors.

## Introduction

Tuberculosis (TB) kills nearly 2 million people annually. The World Health Organization (WHO) declared TB as a global health emergency, which highlights the importance of TB as a major threat to humans [1]. Drug resistance and patient noncompliance are two key factors that affect the success rate of conventional treatments against TB. Therefore, there is an urgent need to identify novel therapeutic targets for TB treatment as well as new drugs that could act on them.

In the last decade, exoenzymes protein tyrosine phosphatase A (PtpA) and B (PtpB) have emerged as promising therapeutic targets to discover new anti-TB agents [2-5]. These enzymes are secreted into the host cell by *Mycobacterium tuberculosis* (Mtb) and attenuate host immune defenses by interfering with the host signaling pathways [6,7]. Thereby, PtpA and PtpB inhibition by small molecules could impact Mtb survival in the host and open the way for the development of innovative therapeutic strategies. Particularly, the localization outside of the mycobacterial cell wall, which is difficult to penetrate, renders these enzymes attractive drug targets.

In previous works we have investigated the inhibitory activity of natural compounds analogues toward PtpA and PtpB from Mtb. In particular, we have first identified potent PtpA inhibitors (IC_50_ = 8.4 - 53.7 μM) by screening a series of naphthylchalcones against this enzyme [8]. Subsequently, we showed that these chalcones inhibit PtpA by means of a competitive and selective mechanism of action (*K*_i_ ranging from 5 to 21 µM) as well as are endowed with a significant inhibitory activity towards Mtb growth in infected macrophages [9]. We have also demonstrated the inhibitory properties of synthetic sulfonyl-hydrazones against PtpB, identified as competitive inhibitors with *K*_i_ values between 2.5 and 15 μM [10]. In our last work, a hundred synthetic chalcones have been investigated for their activities against PtpA and PtpB, and six presented competitive mechanism of action with *K*_i_ values between 8 and 29 μM [11].

In light of recent advances in understanding the pathological involvement of these phosphatases in Mtb growth and proliferation [12], and following our research interest in modulating these enzymes, here we focused on the discovery and characterization of natural compounds as PtpB inhibitors. *In vivo* studies performed with activated macrophages of guinea pigs have shown that gene inactivation of this enzyme provoked accelerated mycobacterial cell death after macrophage invasion [13]. More recently, Zhou and co-workers proposed that PtpB promotes mycobacterial survival in vitro by inhibiting extracellular signal-regulated kinase 1/2 (ERK1/2) and p38 pathways and increasing the phosphorylation of Akt, resulting in reduced production of interleukin-6 (IL-6) and decreased apoptotic activity, respectively [14]. Alber and co-workers have synthesized a strong, competitive and selective PtpB inhibitor, namely OMTS [(oxalylamino-methylene)-thiophene sulfonamide] showing an IC_50_ of 0.44 μM, and solved the three-dimensional structure of the PtpB-inhibitor complex by means of X-ray crystallography [15]. Other groups also have successfully identified inhibitors of PtpB: indole derivatives with selectivity indexes up to 100 [16], cyclic hexapeptides from cyanobacterium *Tychonema* sp. with IC_50_ around 8.0 µM [17], an isoxazole with *K*_i_ value of 0.22 μM [18], selective indolin-2-on-3-spirothiazolidinones with IC_50_ values of 35.5 to 1.2 µM [19] and, recently, benzofurans with sub-micromolar inhibitory activity [20]. Based on these evidences, PtpB has emerged as an important target for anti-TB pharmacological intervention and new inhibitors are in high demand [21].

The screening of natural compounds libraries is a consolidated strategy in drug discovery, which employs the criteria of biological prevalidation and relevance to nature [22]. Natural products have long been recognized as an important source of therapeutically effective agents [23], also because they embody rigid, non-flat three dimensional structures which may positively influence the probability of clinical success of a drug [24]. Indeed, natural products can offer unprecedented opportunities for finding novel hits or leads against a wide range of biological targets. In previous works [8,9,11], we were pioneers in testing libraries of chalcones to find PtpA and PtpB inhibitors, underlining that screening natural products libraries may fuel the discovery of bioactive molecules. Moreover, *in silico* screening is a widely appreciated and reliable tool for prioritizing small molecules for biological testing. Accordingly, in this work we screened *in silico* an *in house* library of natural compounds by means of a structure-based approach composed of molecular docking, rescoring and visual inspection to prioritize few natural compounds as possible PtpB inhibitors that were subsequently assayed *in vitro*. Results of inhibition studies, kinetic measurements and mass spectrometry (MS) assays allowed elucidate their mechanism of action.

## Materials and Methods

### Preparation of the in house library

All compounds of the *in house* library have been previously published and fully characterized. Particularly, compounds studied in this work have been described elsewhere (abbreviations further used in this work are reported in brackets): trachypone (6016) and tetra-acetyl-trachypone (Ac3) [25], Kuwanol E (KuwE) [26], tetra-hydro-isosophoranone (M2H) and isosophoranone (M2) [27], 1,3,8-trihydroxy-6-methyl-4,5,7-triprenylanthrone (PirIII) [28], 4,2’,4’-trimethoxy-6’-hydroxy,3’-prenyl-3-geranyldihydrochalcone (59-triMe) [29], 4-*O*-glucosyl caffeic acid (Caf) [30], 1,3,8-trihydroxy-6-methyl-5,7-diprenyl-4-γ,γ’dihydroxyprenyl-anthrone (Δ3) [31], α-cubebin (α-Cub) [32], bufotenine CH_3_I (Buf-I) [33], 4,2’,4’,6’-tetrahydroxy-3’-prenyl-3-geranyldihydrochalcone (Ega1) [34], cynarin (Cyn) [35] and hesperidin (Hesp) [36] (chemical structures are reported in Figure S3). Most probable tautomeric and ionization states at pH = 7 ± 1 were predicted by the LigPrep application of the Maestro suite [37], and those endowed with a normalized probability higher than 0.7 were retained in the final form of the library. Energy minimization was carried out with the OPLS2005 force field [38].

### Molecular modeling

Coordinates of the target receptor for structure-based molecular modeling were retrieved from the Protein Data Bank, under the accession code PDB ID: 2OZ5 [15]. This structure has been solved by X-ray crystallography at 2.00 Å resolution and represents the only PtpB structure in complex with a small molecular inhibitor which was available at the time of experiments. Coordinates of the protein-ligand complex were energy minimized with Amber11 in a box of explicit TIP3P water molecules (10 Å buffer), by using the ff99bsc0 force field for the protein and the GAFF force field for the OMTS ligand [39,40]. The MM-GBSA python script was used for calculating the delta energy of ligands binding to PtpB, by following a procedure already described [41].

Coordinates of the OMTS were then manually removed and such generated protein structure was used as target receptor during docking calculations with GOLD Program 4.1.2 [42]. The binding site was centered on the CD2 atom of Phe161 and included all PtpB atoms within 20 Å. The highest accuracy of the GOLD genetic algorithm (200%) was used for docking the unique library. The GoldScore function was used. 

The GRID program was used for probing the potential energy of interaction of the OH2 probe atom within the catalytic site of PtpB [43,44]. The Grid center was placed in correspondence of Tyr125 and was of 17.04, 14.40, 16.50 Å (*x*, *y* and *z* axes). Grid maps were then visualized with Ligandscout 3.0 from Inte:ligand [45].

### PTPs expression and purification

PtpB wild type from *Mycobacterium tuberculosis* and human PTP1B wild type expression and purification were done as previously described [11,46].

### Measurement of PtpB inhibition (IC_50_)

The phosphatase assays were carried out similarly as previously described [8,11], in 96-well plates containing 8 µL of diluted compounds in DMSO (final concentration 4%), 20 mM imidazole pH 7.0, 160 µL of MilliQ water and 2 µL of recombinant PtpB (70 ng/µL, in Buffer D - 20 mM Tris–HCl pH 8.0, 50 mM NaCl, 5 mM EDTA, 20% glycerol and 5 mM DTT). The mixture was maintained for 10 min at 37 °C, followed by addition of 20 mM p-nitrophenyl phosphate (pNPP), in order to start the reaction. The enzyme hydrolyzes the substrate (*p*NPP), releasing *p*-nitrophenol. The absorbance was measured with a UV-VIS spectrophotometer (TECAN Magellan Infinite M200) for 10 min at 37 °C (at 410 nm with readings every 1 min). Negative controls were performed in the absence of enzyme or compounds, and positive controls in the presence of enzyme and 4% DMSO. The fraction of residual activity was calculated as the difference in absorbance observed at 2 and 7 minutes of enzyme reaction, obtained by the average of two experiments carried out in triplicate. The IC_50_ values were determined with increasing concentrations of inhibitor (100 nM − 100 μM) versus percentage of residual activity, which was calculated as the difference between the observed absorbance at 2 and 7 min of enzyme reaction, obtained by the average of three independent experiments carried out in triplicate. The experimental data were analyzed with GraphPad Prism 5.0 and the IC_50_ values determined by linear regression. It is important to stress the fact that all compounds are soluble in the assay mixtures at the described experimental conditions. 

### Selectivity assay

The selectivity assays using 2 µL of recombinant PTP1B (120 ng/μL, in buffer B 20 mM bis−Tris pH 6.5, 1 mM EDTA, 3 mM DTT, 10% glycerol, and 92 mM NaCl) were carried out as described above. 

### Enzyme kinetics

To determine the mechanism of inhibition of the compounds, they were screened by the same methodology described before, however, varying concentrations of *p*NPP (at least seven concentrations ranging from 0.2 and 12.8 mM) for each concentration of compound (at least three concentrations ranging from 1 to 40 μM). The reaction rates were expressed as specific activity of the protein (μmol *p*NP.min^−1^.mg^−1^) and the *p*NPP concentration in mM. The *p*-nitrophenol released (1/V) was quantified and analyzed by the Lineweaver−Burk plot (1/[V] × 1/[S]) generated in the GraphPad Prism 5.0. *K*_Mapp_ values obtained for each compound concentration were plotted versus [I], and the intercept of the curve at *x*-axis corresponding to –*K*i. The *K*i values were obtained by the average of at least three independent experiments carried out in triplicate.

### Peptide mass fingerprint analysis

Proteolytic cleavage of recombinant PtpB (5 µM) with sequencing grade modified trypsin (10 μg/ml with a protease:protein ratio of 1:50 (w/w)) (Promega) was performed in 25 mM NH_4_HCO_3_ (pH 7.5) at 37 °C for 3 hours. PtpB was incubated with 300 µM of competitive inhibitor KuwE (100% DMSO) or the same volume of DMSO without inhibitor for 10 min before addition of trypsin. Proteolysis was stopped by homogenizing the sample in the matrix solution of α-cyano-4-hydroxycinnamic acid (5 mg/mL in 50% acetonitrile (ACN) and 0.1% trifluoroacetic acid (TFA)). MS analysis was performed on a MALDI-TOF/TOF spectrometer model Autoflex III (Bruker Daltonics, Bremen, Germany). The spectra generated were analyzed using FlexAnalysis 3.3 sofware (Bruker Daltonics, Bremen, Germany). The experiments are done in quadruplicates.

## Results and Discussion

### Features of the in house library

The *in house* library was obtained from the Organic Chemistry Laboratory of the Dipartimento di Chimica e Tecnologie del Farmaco*, Università di Roma* “La *Sapienza*” (Rome, Italy). This unique library consists of 816 natural products from different classes, mostly flavonoids, benzophenones, xanthones, anthraquinones, ferruginines, alkaloids, steroids, terpenoids, containing different substituents. All molecules have been previously published and fully characterized [25-36]. 

Chemical and physico-chemical features of all compounds were predicted with QikProp [47], to determine reasonable bioavailability as well as drug-like properties. In this respect, MW, LogP, polar surface area (PSA), number of rotatable bonds, hydrogen bond donors (HBD) and acceptors (HBA) were calculated and compared with those of the 95% of commercial drugs. Notably, 92% of compounds have features within the limits set by 95% of commercial drugs, thus emphasizing the drug-likeness of this unique library and its suitability for drug discovery purposes.

After the analysis of possible ionization and tautomeric states at physiological conditions, a sample of 1014 structures from this library was then chosen for docking studies.

### Molecular docking

The ability of some docking programs to reproduce the X-ray determined binding conformation of OMTS within the active site of PtpB was preliminary checked (data not shown). The ligand OMTS was self-docked into the binding site of PtpB whose coordinates were retrieved from the X-ray structure (PDB ID: 2OZ5) [15] and the docked conformation corresponding to the lowest free energy (or highest score) provided by each program was selected as the most probable binding pose. While most programs and functions failed to dock correctly OMTS, the GoldScore function implemented in GOLD [42] provided satisfactory results. In particular, the best agreement between computational and X-ray structural data was obtained by self-docking OMTS toward the receptor structure which was previously energy minimized with Amber11 [39], and including conserved water molecules (RMSD between crystal and docking pose = 0.7441 Å, see also Figure S1). The position and number of water molecules retained within the PtpB catalytic site was established by a GRID analysis performed using the OH2 probe atom (Figure S2). The parameter set used for self-docking OMTS was further used to dock the *in house* unique library within the catalytic site of PtpB. After docking and visual inspection, top 10% ranking compounds were selected for rescoring. 

### Rescoring with MM-GBSA and virtual hits selection

 It is widely appreciated in computer-aided drug design that rescoring docking poses with a scoring function different to that used in generating docking poses could better describe the ligand binding energy toward a receptor [41,48]. In previous studies we evaluated the capability of the Molecular Mechanics Generalized Born Surface Area (MM-GBSA) method in rescoring docking poses generated with GOLD [41,48]. Similar results were discussed also by other research groups [49]. Here, we used the MM-GBSA method for rescoring docking poses of selected molecules and to predict their delta energy of binding (*∆E*). 

Based on the calculated *∆E* and the chemical diversity, fourteen compounds were deemed top priority and selected for biological investigations *in vitro* (docking score and rescoring energy of virtual hits are reported in Table S1).

### Enzymatic assays

**Figure 1:**
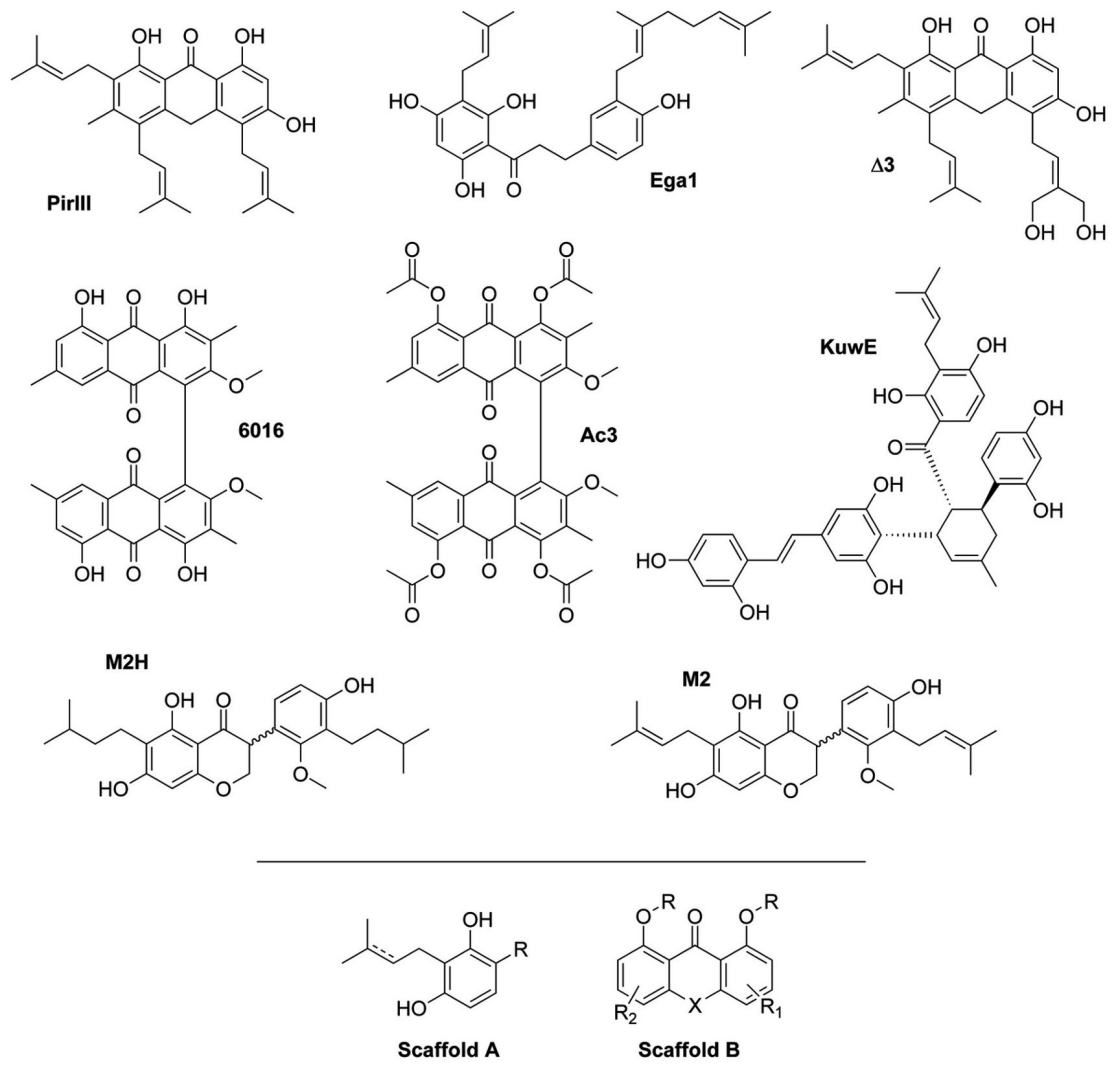
Structures of PtpB inhibitors. Chemical structure of PtpB inhibitors showing an IC_50_ < 100 µM. Below the line are the two common chemical scaffolds: Scaffold A present in KuwE, Ega1, M2 and M2H; Scaffold B present in PirIII, Δ3, 6016 and Ac3.

The inhibitory activity of the fourteen selected natural compounds towards PtpB was evaluated using the previously described methods [8,11], with a slight modification (see Materials and Methods). Six compounds showed significant inhibition of PtpB with IC_50_ < 30µM, two were moderated inhibitors showing IC_50_ between 30 and 100 µM (Figure 1), and six not exhibited enzymatic inhibition at 100µM (Table 1). 

**Table 1:** IC_50_ values of selected hits against PtpB from Mtb.

**Code**	**IC_50_ (µM) PtpB**
**∆3**	26.7 ± 0.6
**PirIII**	5.4 ± 0.6
**KuwE**	1.9 ± 0.5
**Ega1**	13.4 ± 2.6
**M2**	19.8 ± 2.3
**6016**	19.2 ± 6.7
**Hesp**	>100
**Caf**	>100
**Ac3**	33.2 ± 4.9
**59-triMe**	>100
**M2H**	69.4 ± 4.5
**Cyn**	>100
**Buf-I**	>100
**α-Cub**	>100

The results are shown as the average of the individual mean ± SD (standard deviation) for 3 experiments.

The best inhibitory effect of PtpB was achieved by KuwE (IC_50_ = 1.9 ± 0.5 µM), a polyhydroxylated Diels-Alder type adduct metabolite isolated from *Morus nigra* cell cultures [26]. Despite the shortlisted compounds show a significant chemical diversity, the active molecules can be attributed to two distinct molecular scaffolds (Figure 1). Moreover, the comparison of chemical structure of active molecules with their inhibition of PtpB enzymatic activity allowed for delineating rough structure-activity relationships. For instance, comparing anthrone derivatives PirIII (IC_50_ = 5.4 ± 0.6 µM) and ∆3 (IC_50_ = 26.7 ± 0.6 µM), the substitution of two hydrogen atoms with two hydroxyl groups in only one of the prenyl moieties of ∆3, reduced the PtpB inhibitory activity. Based on docking simulations, prenyl groups of PirIII are nicely docked in proximity of a cluster of hydrophobic residues within the PtpB catalytic cavity (Phe98, Leu101, Phe161, Leu199, Ile203, Val231, Leu232) and their hydroxylation such as in ∆3 causes the loss of hydrophobic interactions as well as a different positioning of the tricyclic scaffold in proximity of catalytic residues. The hydrogenation of prenyl groups in the isosophoranone M2H (IC_50_ = 69.4 ± 4.5 µM) reduced the inhibitory potency with respect to M2 (IC_50_ = 19.8 ± 2.3 µM), probably due to the loss of some pi-pi or pi-cation interactions with Arg59, Arg63 and aromatic residues of the PtpB binding site such as Phe133. Moreover, comparing compounds 6016 (IC_50_ = 19.2 ± 6.7 µM) and Ac3 (IC_50_ = 33.2 ± 4.9 µM), the acetylation of the four hydroxyl groups in Ac3 provides a steric hindrance that, by docking, determines a different orientation of Ac3 within the PtpB catalytic cavity, leading to a slight loss of inhibitory activity in vitro. By comparing dihydrochalcones 59-triMe and Ega1, in spite of the different position of the prenyl and geranyl groups, the methylation of three hydroxyl groups in 59-triMe (IC_50_ >100 µM) reduced drastically the inhibitory activity with respect to Ega1 (IC_50_ = 13.4 ± 2.6 µM) due to the loss of H-bond interactions with a conserved water molecule and the protein backbone within Phe161 and Ala162. In summary, we found that the methylation or acetylation of hydroxyl groups in dihydrochalcones and trachypones, respectively, as well as the hydrogenation of prenyl groups in isosophoranones reduced significantly the PtpB inhibitory activity, whereas the presence of non-hydroxylated prenyl groups seems to be very important for the activity of anthrones. Other mono or polyhydroxylated compounds were not active (Hesp, Caf, Cyn, Buf-I and α-Cub). 

Overall, we found a satisfactory correlation between rescoring energy and enzymatic inhibition data of active compounds(-logIC_50_), with the only exception of ∆3 whose binding affinity was overestimated by both docking and rescoring. By removing the outlier ∆3, we found a R^2^ correlation value of 0.23 by comparing the –logIC_50_ values with docking scores and a R^2^ of 0.73 by comparing rescoring energies (Figure S5), thus reinforcing the robustness of our computational approach as well as that rescoring docking poses with the MM-GBSA may provide a higher correlation with experimental data than docking.

It is worth noting that the presence of a carboxyl group in OMTS seems to be essential for the interactions with PtpB catalytic site [15] but, contrarily to expectation, compounds Caf and Cyn endowed with the carboxyl group were not active in our assays.

### Kinetics measurements

**Figure 2:**
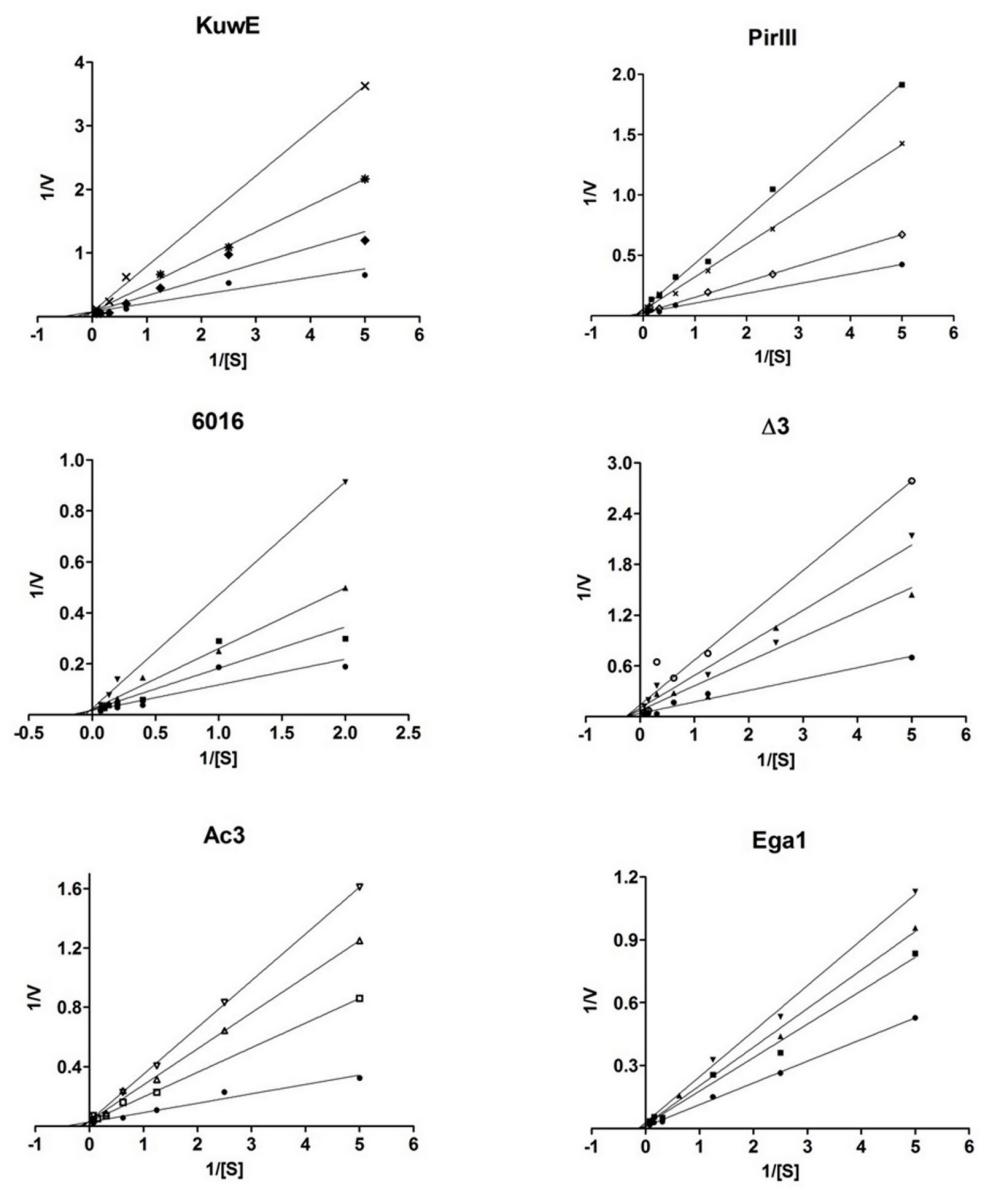
Kinetics measurements of PtpB inhibitors. Lineweaver-Burk double-reciprocal plots representing inhibitory profiles of compounds KuwE, PirIII, Ega1, 6016, Ac3 and ∆3 against PtpB. Kinetic experiments were conducted in the presence of increasing concentrations of inhibitors: 0 µM (), 1 µM (), 2 µM (), 3 µM (), 6 µM (), 10 µM (), 20 µM (), 25 µM (), 30 µM (), 35 µM (), 40 µM (), 45 µM (); *p*NPP was used as substrate in all experiments. For KuwE, Ac3 and 6016, all lines converged at the *y*-axis (1/*V*_max_), whereas the slope (K_Mapp_/*V*_max_) and *x*-axis interception (1/*K*_Mapp_) varies according to the inhibitor concentration; the constant value of *V*_max_ and the increased values of *K*_Mapp_ are consistent with a competitive inhibition mechanism. For PirIII, Ega1 and Δ3, all lines converge at the *x*-axis (1/*K*_Mapp_) and the *y*-axis interception (1/*V*_max_) varies as a function of the inhibitor concentration; the constant value of *K*_Mapp_ and the increased values of *V*_max_ indicate that these compounds are noncompetitive inhibitors.

Most potent PtpB inhibitors were then selected to investigate their mechanism of action with respect to the PTPs substrate p-nitrophenyl phosphate (pNPP). Kinetic analysis revealed that compounds KuwE, 6016 and Ac3 act as PtpB competitive inhibitors, with *K*_i_ values between 1.6 and 17.1 µM, while PirIII, Ega1 and ∆3 are non-competitive inhibitors, with *K*_i_ values between 6.6 and 14.5 µM (Table 2). Figure 2 shows the Lineweaver-Burk plots of the PtpB inhibitors. 

**Table 2:** *K*_i_ values, IC_50_/*K*_i_ ratio and type of inhibition of the PtpB inhibitors.

**Compound**	**PtpB *K*_i_ (µM)**	**IC_50_ / *K*_i_**	**Type of inhibition**
**KuwE**	1.6 ± 0.1	1.2	Competitive
**PirIII**	6.6 ± 2.7	0.8	Non-competitive
**6016**	11.5 ± 1.7	1.7	Competitive
**∆3**	13.4 ± 1.5	2.0	Non-competitive
**Ega1**	14.5 ± 2.3	0.9	Non-competitive
**Ac3**	17.1 ± 4.5	1.9	Competitive

*K*_i_ values are shown as the average of the individual mean ± SD (standard deviation).

### Selectivity assays

**Figure 3:**
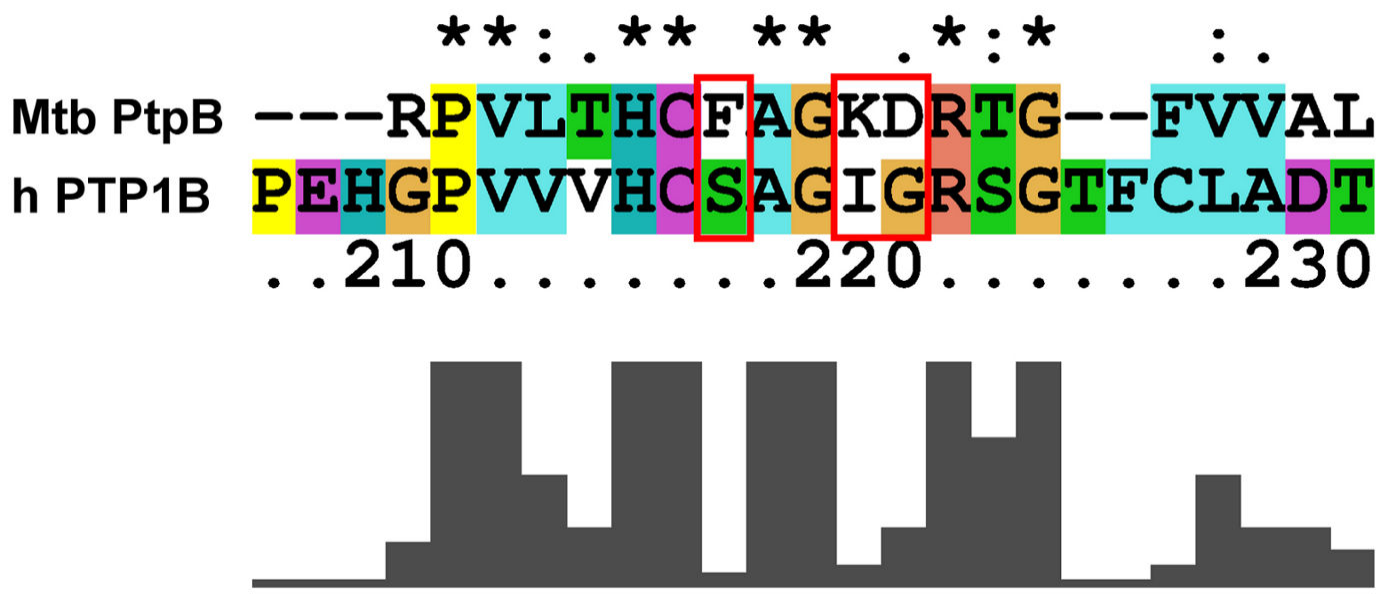
Differences between PtpB and PTP1B. Sequence alignment of Mtb PtpB (UniProtKB code: P96830, 276 aa complete sequence) and human PTP1B (UniProtKB code: P18031, 435 aa complete sequence). Not conserved amino acids of the PtpB active site motif, which may be exploited to design selective Mtb PtpB inhibitors, are highlighted by a red box. Sequence alignment was performed with ClustalX. Sequence numbering corresponds to human PTP1B. Bars below the sequence alignment correspond to the degree of amino acid conservation between the two sequence (full bar: residues identity; empty bar: completely different residues).

Human protein tyrosine phosphatase 1B (PTP1B) plays a critical role in regulating glucose homeostasis and body weight by acting as a key negative regulator of insulin and leptin signaling pathway, respectively [50]. This enzyme has been shown to increase insulin sensitivity and obesity resistance [51]. The tyrosine phosphatase family shares a catalytic domain with the conserved invariant sequence HCX_5_R [52,53]. Especially, PtpB (H159C160X_5_R166) has a Phe161, a Lys164 and an Asp165 that are conserved and differ from human PTP1B (H214C215X_5_R221) (Figure 3) and might be therefore exploited to design inhibitors with improved selectivity.

The most potent PtpB inhibitors were evaluated against the human PTP1B (Table 3), in order to monitor their selectivity index (SI) as the ratio between IC_50_ measured towards human PTP1B and PtpB from Mtb. Results showed that the most potent compounds, namely KuwE, PirIII and Ega1 are slightly selective for PtpB, with a SI of 5.1, 2.3 and 1.6, respectively. Other compounds exhibited a SI lower than 1. 

**Table 3:** IC_50_ values of most potent PtpB inhibitors towards PtpB from Mtb and human PTP1B, and selectivity index (SI).

**Code**	**IC_50_ (µM) PtpB**	**IC_50_ (µM) PTP1B**	SI*
**∆3**	26.7 ± 0.6	14.7 ± 2.1	0.6
**PirIII**	5.4 ± 0.6	11.8 ± 3.5	2.3
**KuwE**	1.9 ± 0.5	9.6 ± 2.6	5.1
**Ega1**	13.4 ± 2.6	20.9 ± 2.8	1.6
**6016**	19.2 ± 6.7	7.1 ± 1.4	0.4
**Ac3**	33.2 ± 4.9	31.2 ± 2.7	0.9

The results are shown as the average of the individual mean ± SD (standard deviation) for 3 experiments. * SI (Selectivity index), given by (IC_50_^PTP1B^/IC_50_^PtpB^).

### Predicted binding mode of KuwE

**Figure 4:**
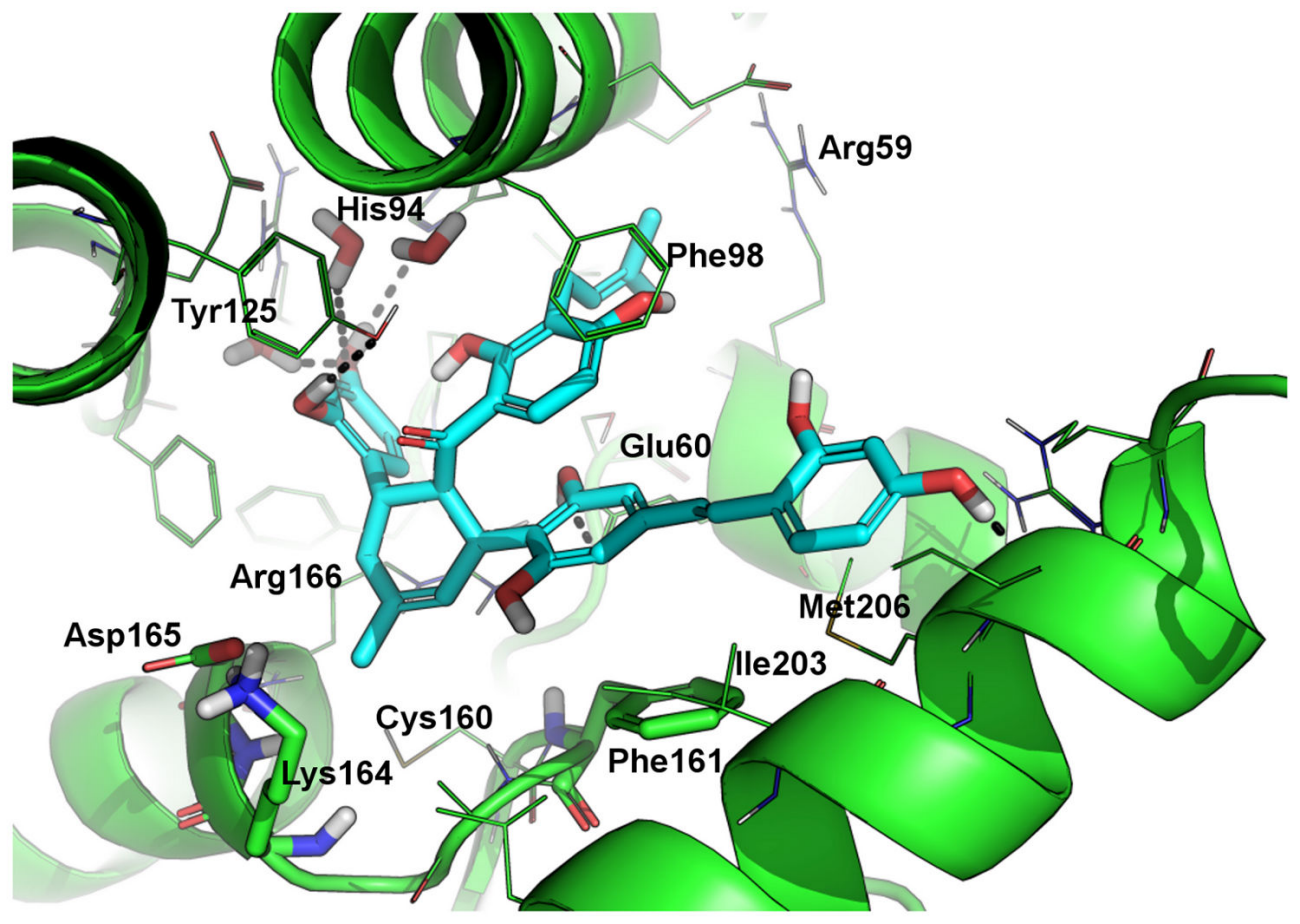
Docking-based binding mode of KuwE. The binding mode of KuwE within the catalytic site of PtpB, as predicted by docking. KuwE is shown as cyan sticks; PtpB is represented as green cartoon and lines. Polar contacts between KuwE and PtpB are highlighted as black dotted lines. Residues Phe161, Lys164 and Asp165 that are not conserved in the human PTP1B are showed as green sticks. Residues numbering follows the PDB: 2OZ5 numbering scheme.

The possible binding mode of KuwE within the catalytic site of PtpB was investigated by molecular docking (Figure 4). The hydroxyl groups of KuwE perform H-bond interactions with Glu60, His94, Tyr125, Met206 and two conserved water molecules. Only the H-bond with His94 and water molecules are in common with the binding of OMTS revealed by X-ray crystallography, although there is a clear shape overlapping between these inhibitors within the PtpB active site. KuwE aromatic rings belonging to the dihydroxyphenyl-vinyl moiety are involved in hydrophobic interactions with a cluster of PtpB hydrophobic residues such as Ile203, Met206, Ile207 and Phe161 located at the entrance of the catalytic site, while the prenyl group shows a fine overlapping with a phenyl ring of OMTS and binds in a hydrophobic sub-pocket in close proximity of Leu83.

Notably, KuwE performs a hydrophobic/aromatic interaction with the side chain of the non-conserved Phe161 (Figure 4) with geometry resembling the parallel displaced π-stacking interaction, thus providing a possible structural explanation for KuwE selectivity observed by enzymatic assays. Such structural feature could be exploited for the rational design of selective PtpB inhibitors as well as to improve the selectivity of already known PtpB inhibitors.

### Peptide mass fingerprint analysis

**Figure 5:**
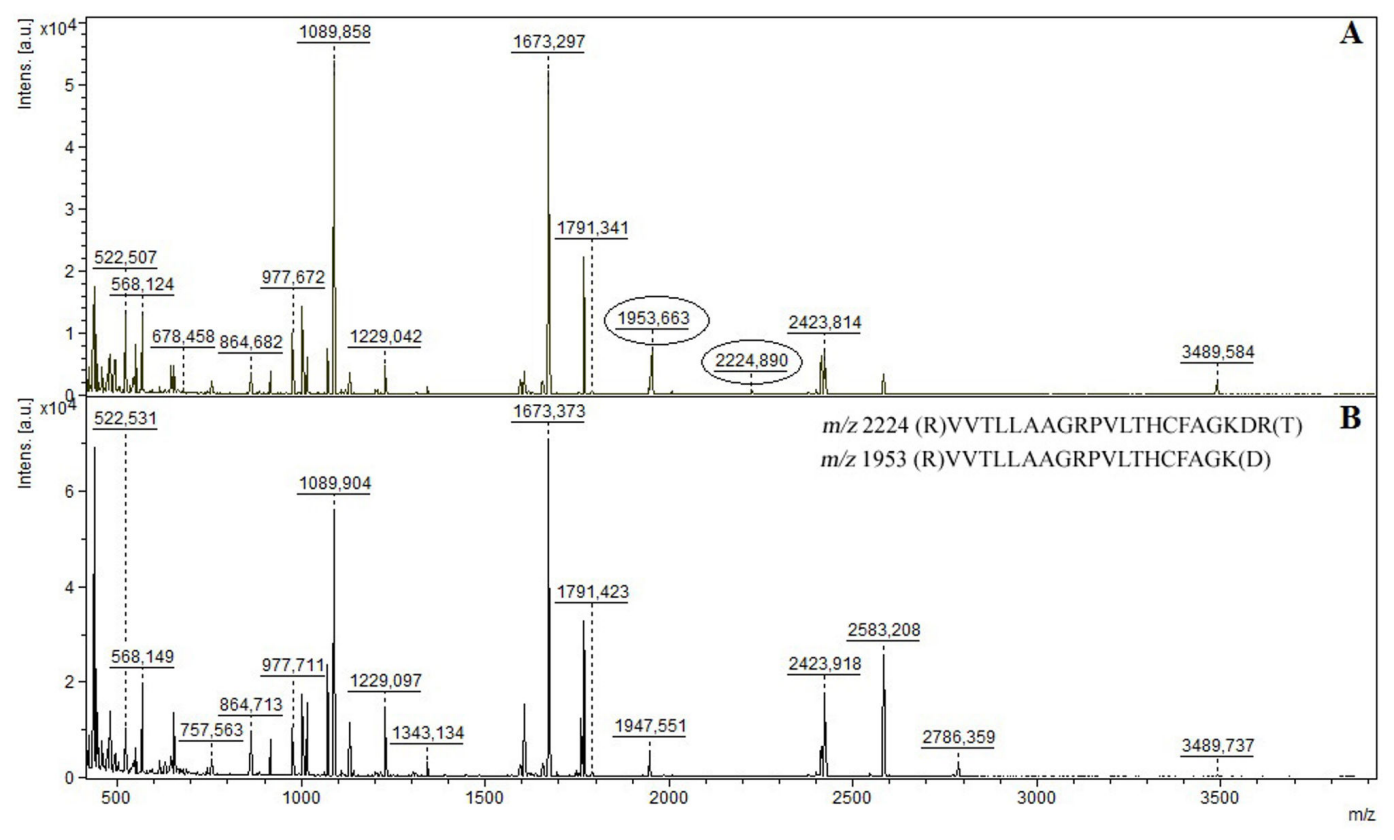
Peptide mass fingerprints. Peptide mass fingerprinting of PtpB recorded by MS in absence (A) and presence of 300 µM KuwE (B). The tryptic peptide *m/z* 2224 corresponds to the complete sequence of the catalytic site ((R145)VVTLLAAGRPVLTHCFAGKDR(T167)) and the tryptic peptide *m/z* 1953 corresponds to a part of the catalytic site ((R145)VVTLLAAGRPVLTHCFAGK(D165)), which include the His159 and the catalytic Cys160 residues.

**Figure 6:**
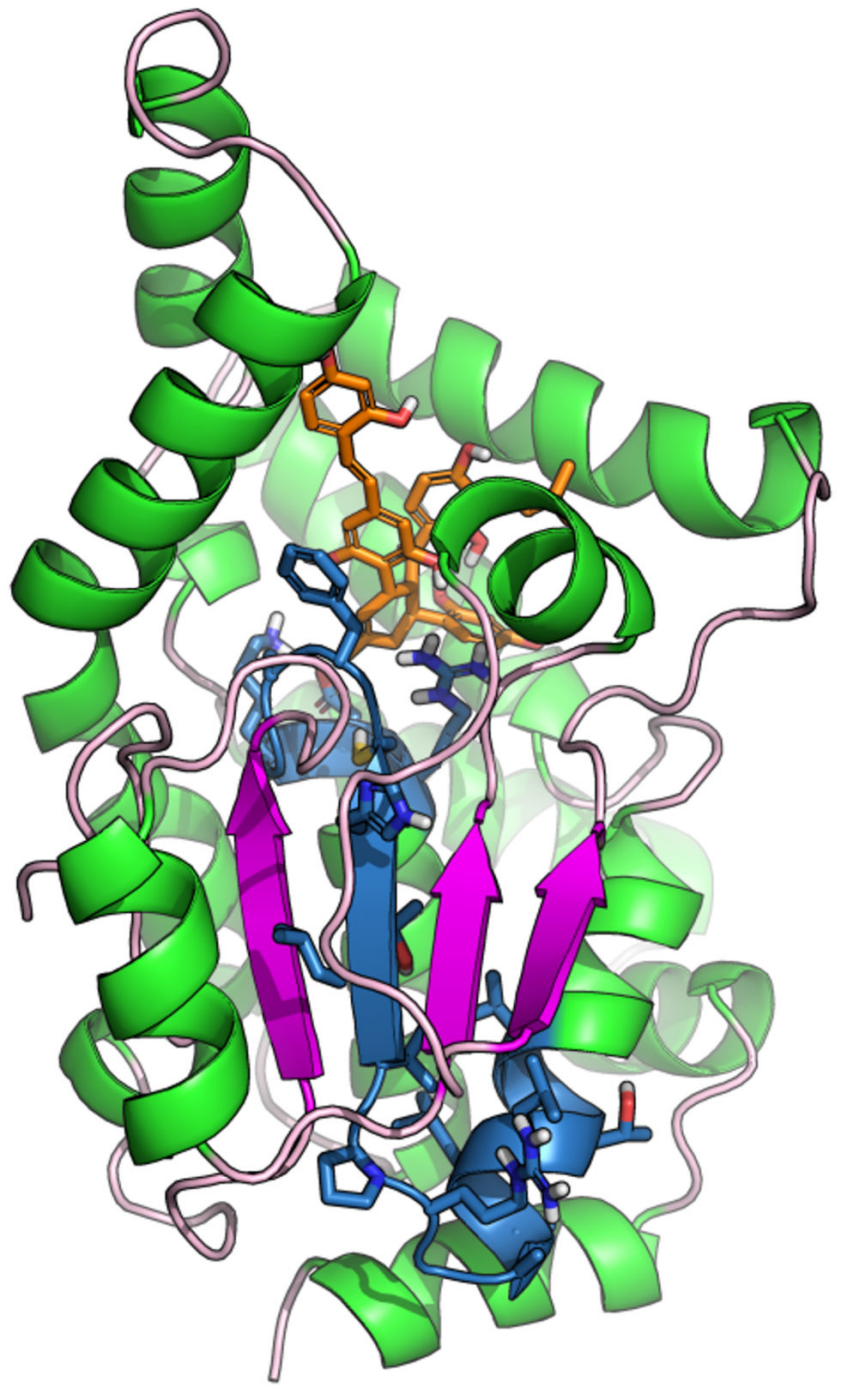
Protection of PtpB proteolytic cleavage by KuwE. Schematic representation of the region protected by KuwE in PtpB structure. The amino acid sequence VVTLLAAGRPVLTHCFAGKDR (*m/z* 2224) identified by mass spectrometry is highlighted in blue sticks and cartoon. KuwE is showed as orange sticks while PtpB is represented as green (alpha-helix) and magenta (beta-chain) cartoon.

To monitor the capability of KuwE to protect the PtpB catalytic site from the proteolytic cleavage by trypsin, as well as to support the proposed mechanism of inhibition, the peptide mass fingerprint (PMF) of PtpB in presence or absence of inhibitor was determined by mass spectrometry (MS). The sequence coverage by MS analysis of the tryptic digest of PtpB was 78% and 81%, respectively, in the presence or absence of KuwE. By comparing the PtpB PMF in the absence (Figure 5A) and presence (Figure 5B) of KuwE, a significant difference in fragment composition was observed. In fact, when PtpB proteolysis was carried out in the absence of KuwE, two fragments (*m/z* 1953 and *m/z* 2224) were observed, which are not present in the mass spectrum recorded in the presence of 300 µM of KuwE. Since KuwE is not able to inhibit trypsin by itself, these results suggest that KuwE inhibits the formation of the cleavage products of 1953 Da and 2224 Da by interacting within the PtpB catalytic site and protecting it from the proteolytic cleavage by trypsin. The peptide with *m/z* 2224 corresponds to the tryptic fragment of the complete sequence of the catalytic site ((R145)VVTLLAAGRPVLTHCFAGKDR(T167)) (Figure 6), while the fragment with *m/z* 1953 corresponds to a part of the catalytic site with sequence ((R145)VVTLLAAGRPVLTHCFAGK(D165)), which include the His159 and the catalytic Cys160 residues. Notably, these evidences are in agreement with docking results showing that KuwE interacts within the PtpB catalytic site in close proximity of Cys160, Phe161, Lys164 and Arg166 which are present in the fragment cleaved by trypsin in absence of the inhibitor. 

Furthermore, we monitored also the effect of the PtpB competitive inhibitor 70 [11] used as positive control, towards the trypsin proteolytic cleavage of PtpB. Results showed that this molecule is also able at least to reduce significantly the intensity of peaks *m/z* 1953 and *m/z* 2224 in PMF spectra recorded in presence of PtpB and trypsin (Figure S4). 

In summary, analysis of PMF suggests that KuwE may protect PtpB cleavage by trypsin by interacting and shielding the catalytic site. Indeed, the tryptic fragments were not detected in the PMF of PtpB recorded in presence of the competitive inhibitor KuwE. These results also support the interaction model proposed by molecular modeling for KuwE, which is shown in Figure 4.

## Conclusions

In this work we reported on the discovery of natural compounds as potent inhibitors of PtpB by *in silico* screening of an *in house* unique library and enzymatic assays. Especially, KuwE showed the most potent inhibition (*K*_i_ = 1.6 ± 0.1 µM) and, to the best of our knowledge, KuwE is the most potent natural compound inhibitor of PtpB from Mtb discovered so far, as well as this is the first report on a non-peptidic natural compound inhibitor of PtpB. Kinetic studies shed light on the mechanism of PtpB inhibition, whereas peptide mass fingerprint analyses performed by MS showed that KuwE was able to protect the PtpB catalytic site by the proteolytic activity of trypsin, thus reinforcing that this inhibitor may interact within the PtpB catalytic site, in agreement with docking results. Despite the success in the identification of natural compounds as potent inhibitors of PtpB, a low selectivity towards the human PTP1B was found. In this respect, the rational optimization of KuwE should account for challenging organic synthesis strategies. 

Natural products continue to represent a unique source of chemical diversity for the discovery of hit and lead molecules. PtpB natural product inhibitors discovered and characterized in this study may serve as profitable tool to investigate the biochemical functions of PtpB as well as starting point for further optimization aimed at the development of anti-TB medicinally active agents.

## Supporting Information

Figure S1 **Self-docking of OMTS.** Amber minimization and GoldScore docking: the best superimposition between generated structure (magenta) and X-ray complex (cyan).
(TIF)

Figure S2 **Water molecules into the active site of PtpB.** Crystallographic water molecules are showed as red spheres; GRID-generated potentials for WAT probe are showed as light yellow meshes.
(TIF)

Figure S3 **Chemical structure of natural compounds selected by virtual screening.**
(TIF)

Figure S4 **Peptide Mass Fingerprint of PtpB in absence (top) and presence (below) of 70 at 300 µM.** The tryptic peptide *m/z* 2224 corresponds to the complete sequence of the catalytic site ((R145)VVTLLAAGRPVLTHCFAGKDR(T167)) and the tryptic peptide *m/z* 1953 corresponds to a part of the catalytic site ((R145)VVTLLAAGRPVLTHCFAGK(D165)), which include the His159 and the catalytic Cys160 residues.
(TIF)

Figure S5 **Plot of rescoring energy calculated with the MM-GBSA method versus –LogIC_50_ of active compounds, measured *invitro*.** The values of ∆3 were removed (outlier).
(TIF)

Table S1 **Docking score and rescoring energy of natural compounds selected from virtual screening as possible PtpB inhibitors.**
(DOCX)

Text S1 **Additional information on molecular modeling directions and Peptide mass fingerprint analysis.**
(DOCX)
