# Supplementary figures and images for: Discovery of Mycobacterium tuberculosis Protein Tyrosine Phosphatase B (PtpB) Inhibitors from Natural Products
Source: PLoS One. 2013 Oct 14;8(10):e77081. doi: 10.1371/journal.pone.0077081 (PMC3796549; doi:10.1371/journal.pone.0077081)

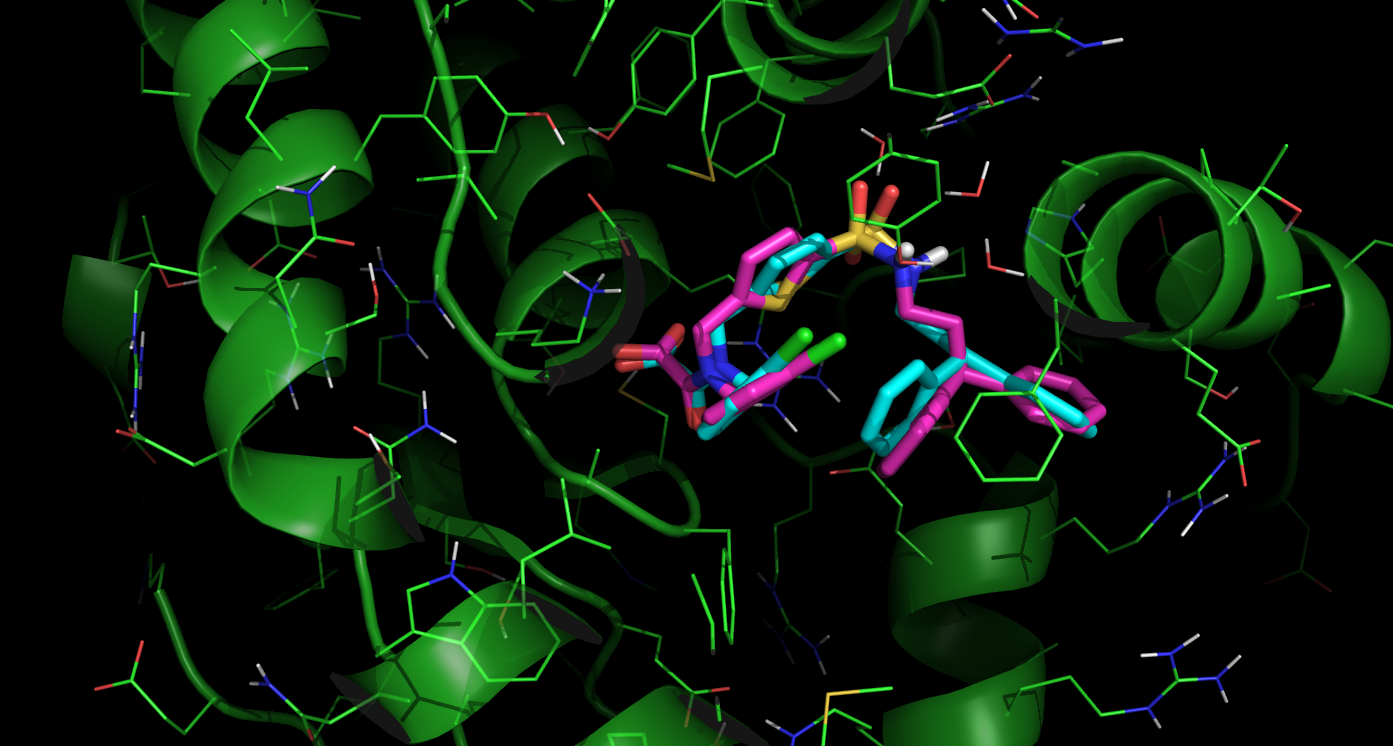

Supplement: Figure S1 — Self-docking of OMTS. Amber minimization and GoldScore docking: the best superimposition between generated structure (magenta) and X-ray complex (cyan). (TIF) [file pone.0077081.s001.tif]

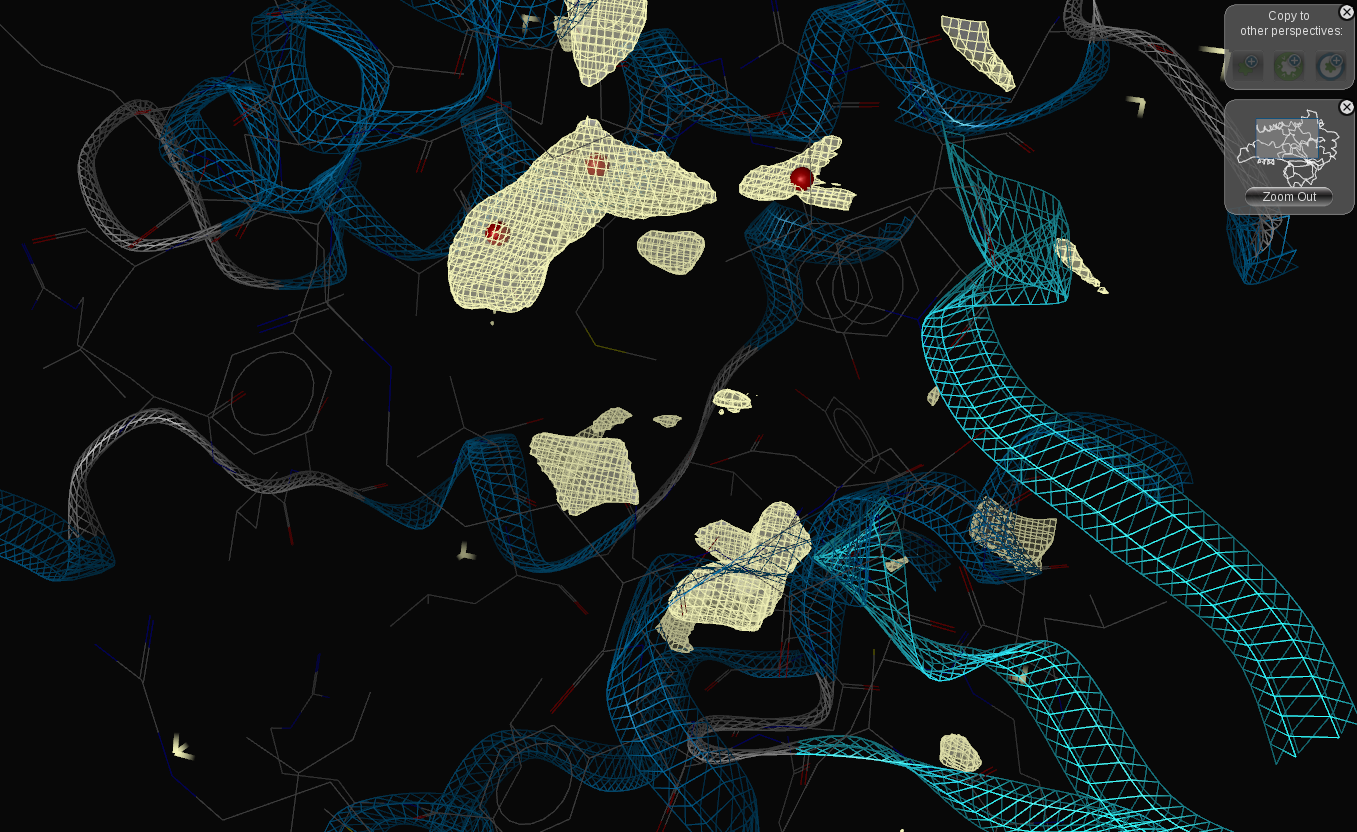

Supplement: Figure S2 — Water molecules into the active site of PtpB. Crystallographic water molecules are showed as red spheres; GRID-generated potentials for WAT probe are showed as light yellow meshes. (TIF) [file pone.0077081.s002.tif]

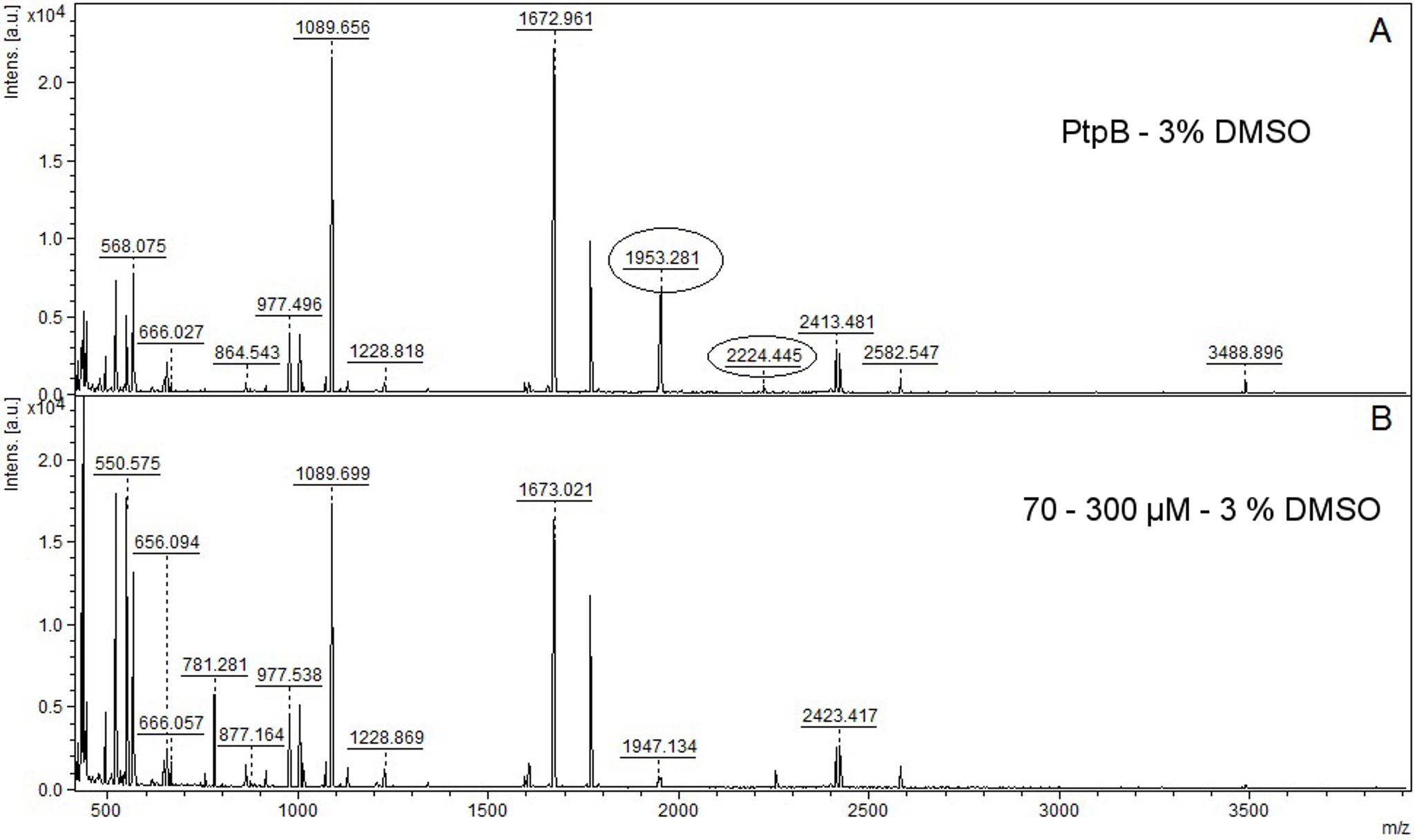

Supplement: Figure S4 — Peptide Mass Fingerprint of PtpB in absence (top) and presence (below) of 70 at 300 µM. The tryptic peptide m/z 2224 corresponds to the complete sequence of the catalytic site ((R145)VVTLLAAGRPVLTHCFAGKDR(T167)) and the tryptic peptide m/z 1953 corresponds to a part of the catalytic site ((R145)VVTLLAAGRPVLTHCFAGK(D165)), which include the His159 and the catalytic Cys160 residues. (TIF) [file pone.0077081.s004.tif]

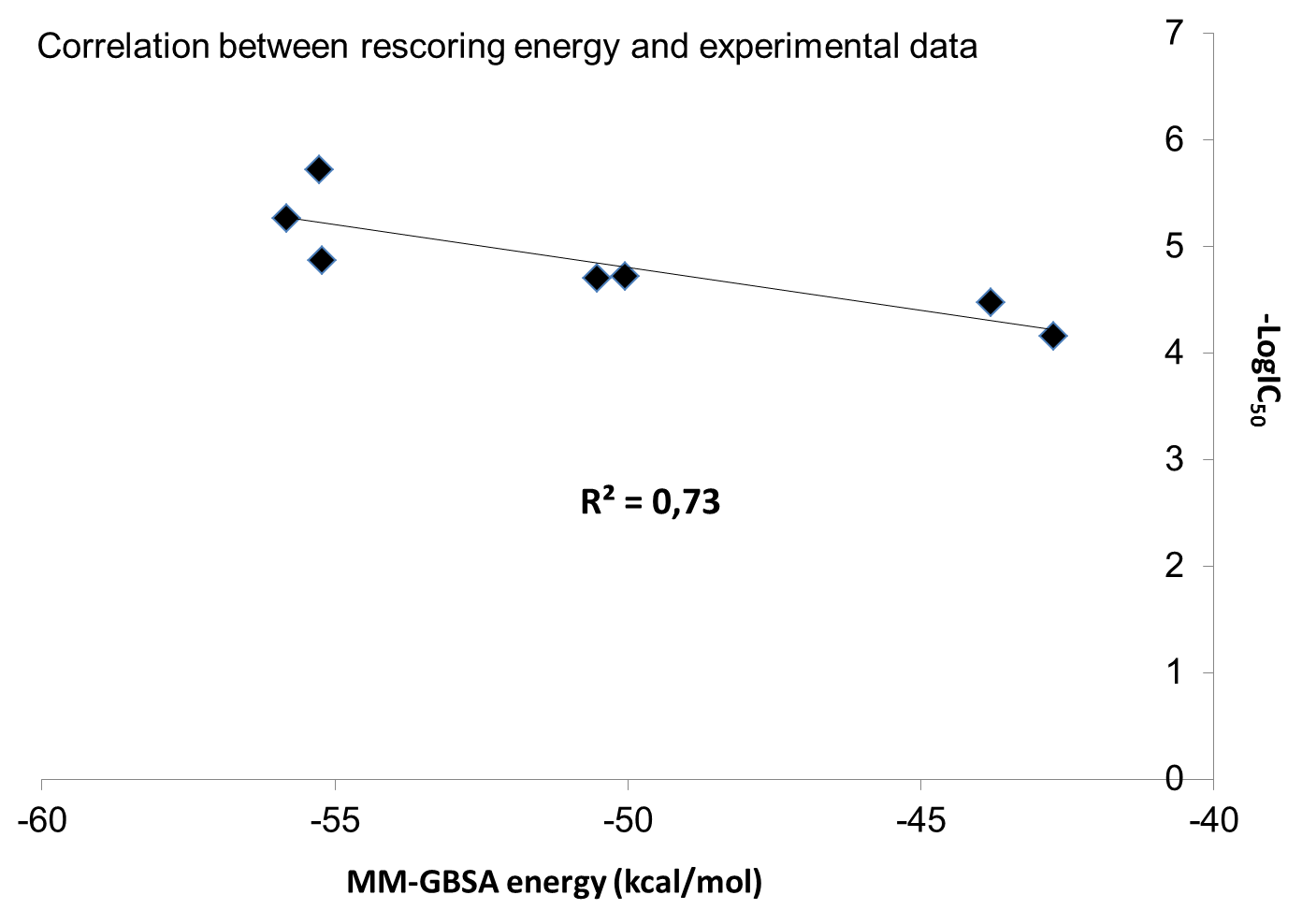

Supplement: Figure S5 — Plot of rescoring energy calculated with the MM-GBSA method versus –LogIC50 of active compounds, measured invitro. The values of ∆3 were removed (outlier). (TIF) [file pone.0077081.s005.tif]
