# Supplementary material for: Discovery of Mycobacterium tuberculosis Protein Tyrosine Phosphatase B (PtpB) Inhibitors from Natural Products
Source: PLoS One. 2013 Oct 14;8(10):e77081. doi: 10.1371/journal.pone.0077081 (PMC3796549; doi:10.1371/journal.pone.0077081)
Supplement: Table S1 — Docking score and rescoring energy of natural compounds selected from virtual screening as possible PtpB inhibitors. (DOCX) [file pone.0077081.s006.docx]

| **Code** | **Compound name** | **Docking (Goldscore Fitness)** | **Rescoring*(MM-GBSA)^#^ ± SD** |  |
| --- | --- | --- | --- | --- |
| **∆3** | 1,3,8-trihydroxy, 6-methyl, 5,7-diprenyl, 4-γ,γ’dihydroxyprenyl-anthrone | 67.09 | -62.31 ± 1.09 |  |
| **PirIII** | 1,3,8-trihydroxy, 6-methyl, 4,5,7-triprenyl-anthrone | 66.75 | -55.83 ± 2.08 |  |
| **KuwE** | Kuwanol E | 68.78 | -55.26 ± 1.48 |  |
| **Ega1** | 4,2’,4’,6’-tetrahydroxy,3’-prenyl,3-geranyl-dihydrochalcone | 69.28 | -55.21 ± 1.59 |  |
| **M2** | Isosophoranone | 68.46 | -50.53 ± 1.96 |  |
| **6016** | Trachypone | 72.02 | -50.04 ± 1.83 |  |
| **Hesp** | Hesperidin | 70.08 | -47.85 ± 3.81 |  |
| **Caf** | 4-*O*-glucosyl caffeic acid | 67.55 | -44.79 ± 2.00 |  |
| **Ac3** | Tetra-acetyl-trachypone | 73.94 | -43.81 ± 1.51 |  |
| **59-triMe** | 4,2’,4’-trimethoxy,6’-hydroxy,3’-prenyl,3-geranyl-dihydrochalcone | 79.38 | -43.11 ± 4.13 | |
| **M2H** | Tetra-hydro-isosophoranone | 69.37 | -42.73 ± 1.26 | |
| **Cyn** | Cynarin | 79.03 | -42.14 ± 5.57 |  |
| **Buf-I** | Bufotenine CH_3_I | 55.00 | -33.02 ± 0.48 |  |
| **α-Cub** | α-cubebin | 76.88 | -32.18 ± 6.12 |  |

**Table S1.** Docking score and rescoring energy of natural compounds selected from virtual screening as possible PtpB inhibitors.

^#^ MM-GBSA energy is expressed in kcal/mol. SD is conformational standard deviation. *Results are sorted by increasing rescoring energy.
