# Supplementary material for: Discovery of Mycobacterium tuberculosis Protein Tyrosine Phosphatase B (PtpB) Inhibitors from Natural Products
Source: PLoS One. 2013 Oct 14;8(10):e77081. doi: 10.1371/journal.pone.0077081 (PMC3796549; doi:10.1371/journal.pone.0077081)
Supplement: Text S1 — Additional information on molecular modeling directions and Peptide mass fingerprint analysis. (DOCX) [file pone.0077081.s007.docx]

**Supporting Information Text**

**Discovery of *Mycobacterium tuberculosis* protein tyrosine phosphatase B (PtpB) inhibitors from natural products**

Alessandra Mascarello, Mattia Mori, Louise Domeneghini Chiaradia-Delatorre, Angela Camila Orbem Menegatti, Franco Delle Monache, Franco Ferrari, Rosendo Auguto Yunes, Ricardo José Nunes, Hernán Terenzi, Bruno Botta, Maurizio Botta

**1. Docking settings**

The highest agreement between OMTS docking-based binding mode and the X-ray-based binding mode were obtained by docking OMTS with the GoldScore function of GOLD (version 4.1.2) toward a receptor structure which was energy minimized by means of Amber11 (RMS = 0.7741 Å) (Figure S1).

**2. Role of crystal water molecules**

Based on the only crystal structure available for describing the complex between PtpB and a small molecular inhibitor (PDB ID: 2OZ5) we first examined the role of crystal water molecules located within the co-crystalized ligand, namely OMTS (~1.8 Å distance) [[1](#_ENREF_1)s]. In fact, although for many years explicit water molecules were not accounted for during molecular docking studies, it has been reported that including conserved waters could significantly improve docking results [[2](#_ENREF_2)s,[3](#_ENREF_3)s]. Here, the GRID program [[4](#_ENREF_4)s,[5](#_ENREF_5)s] was used to explore possible sites for water binding into the active site of PtpB. GRID maps were generated by using the WAT probe. A fine overlapping between minimum energy regions and the coordinates of three crystallographic water molecules which are trapped in the PtpB active site was observed (Figure S2). Since these water molecules are in close proximity of the OMTS and seem to bridge the sulfonamide moiety with PtpB, one may speculate they could stabilize the complex and mediate protein-ligand interactions, adding a favorable entropic contribution to the ligand free energy of binding. For this reason, and according to GRID calculations, these water molecules were included in our receptor structure and explicitly considered during docking. It is worth noting that other studies have reported the improvement of docking results by docking with GOLD, when considering an average of three or four water molecules involved in bridging ligand with protein [[6](#_ENREF_6)s].

**3. Docking and rescoring results**

Results obtained from docking and rescoring studies are summarized in Table S1 and clearly indicate that compounds Δ3, PirIII, KuwE, Ega1, M2 and 6016 (Figure S3) might have higher affinity for PtpB than other compounds. The ranking order obtained by docking is significantly different than that after rescoring (Table S1), thus stressing again that rescoring docking poses might have a large impact on the enrichment rate provided by a virtual screening protocol. Comparison with experimental affinity data discussed below emphasizes the quality of MM-GBSA in rescoring docking poses generated with GOLD.

**5. Peptide mass fingerprint analysis of PtpB natural compounds inhibitors**

Inhibitor: **70** – positive control (competitive, IC_50_ = 12.0 ± 2.0 µM; *K*_i_ = 8.0 ± 1.0 µM) [[7](#_ENREF_7)s], see Figure S4

**References**

1s. Grundner C, Perrin D, Hooft van Huijsduijnen R, Swinnen D, Gonzalez J, et al. (2007) Structural basis for selective inhibition of Mycobacterium tuberculosis protein tyrosine phosphatase PtpB. Structure 15: 499-509.

2s. Santos R, Hritz J, Oostenbrink C (2010) Role of water in molecular docking simulations of cytochrome P450 2D6. J Chem Inf Model 50: 146-154.

3s. Cappel D, Wahlstrom R, Brenk R, Sotriffer CA (2011) Probing the dynamic nature of water molecules and their influences on ligand binding in a model binding site. J Chem Inf Model 51: 2581-2594.

4s. Carosati E, Sciabola S, Cruciani G (2004) Hydrogen bonding interactions of covalently bonded fluorine atoms: from crystallographic data to a new angular function in the GRID force field. J Med Chem 47: 5114-5125.

5s. Goodford PJ (1985) A computational procedure for determining energetically favorable binding sites on biologically important macromolecules. J Med Chem 28: 849-857.

6s. de Beer SB, Vermeulen NP, Oostenbrink C (2010) The role of water molecules in computational drug design. Curr Top Med Chem 10: 55-66.

7s. Chiaradia LD, Martins PG, Cordeiro MN, Guido RV, Ecco G, et al. (2012) Synthesis, biological evaluation, and molecular modeling of chalcone derivatives as potent inhibitors of Mycobacterium tuberculosis protein tyrosine phosphatases (PtpA and PtpB). J Med Chem 55: 390-402.

8s. Madhurantakam C, Chavali VR, Das AK (2007) Analyzing the catalytic mechanism of MPtpA: A low molecular weight protein tyrosine phosphatase from Mycobacterium tuberculosis through site-directed mutagenesis. Proteins 71: 706-714.
